# Gambling-Like Day Trading During the COVID-19 Pandemic – Need for Research on a Pandemic-Related Risk of Indebtedness and Mental Health Impact

**DOI:** 10.3389/fpsyt.2021.715946

**Published:** 2021-07-26

**Authors:** Anders Håkansson, Fernando Fernández-Aranda, Susana Jiménez-Murcia

**Affiliations:** ^1^Department of Clinical Sciences Lund, Psychiatry, Faculty of Medicine, Lund University, Lund, Sweden; ^2^Region Skåne, Malmö Addiction Center, Clinical Sports and Mental Health Unit, Malmö, Sweden; ^3^Department of Psychiatry, Bellvitge University Hospital-L'Institut d'Investigació Biomèdica de Bellvitge (IDIBELL), Barcelona, Spain; ^4^Ciber Fisiopatología Obesidad y Nutrición (CIBERObn), Instituto de Salud Carlos III, Madrid, Spain; ^5^Department of Clinical Sciences, School of Medicine and Health Sciences, University of Barcelona, Barcelona, Spain

**Keywords:** gambling, problem gambling, COVID-19, day trading, stock exchange

## Abstract

Stock exchange trading increasingly has been highlighted as a possible cause of gambling disorder, typically in rapid and excessive “day trading” which may cause over-indebtedness and mental health problems. The COVID-19 pandemic has been suspected to increase online gambling and gambling problems. In a number of recent media reports, day trading has been reported to increase during COVID-19, possibly in relation to changes in everyday life, financial problems and job insecurity during the pandemic. Increasing day trading has thereby been suspected to cause addictive behavior, financial difficulties, and poor mental health. However, there is hitherto a lack of research in the area. The present paper addresses the potential for day trading to cause problem gambling, debts and mental health problems, and calls for research and clinical guidelines in problem gambling related to stock market behavior as a problematic gambling behavior. Screening tools, awareness among clinicians, and longitudinal research studies may be warranted, both during the COVID-19 pandemic and beyond.

## Introduction

Poor mental health has been raised as one of the public health concerns related to the COVID-19 pandemic (1, 2). Gambling disorder, an established addictive disorder involving excessive and problematic gambling for money, is known to impact mental health in those affected and among their families (3, 4). Increased gambling practices, potentially becoming addictive and leading to a gambling disorder, is one of the public health challenges mentioned as a possible consequence of the COVID-19 pandemic (5).

Problem gambling, with or without the progression into a gambling disorder (3), is known to cause debts and over-indebtedness in affected individuals and for those living in their households. This is also known to cause severe mental health consequences in those affected (6). It is also known that indebtedness in general is a risk factor of a number of medical consequences (7), including a link to suicidal behavior (8).

Over the past few years, stock exchange trading has been increasingly highlighted as one of the gambling types – or as a gambling equivalent – occurring in a minority of treatment-seeking gambling disorder patients. In recent years, for example, authors described that around eight percent of financial market investors may fulfill criteria of problem gambling in relation to their trading behavior, and for whom around half of them may fulfill criteria of a gambling disorder (9). It has been argued that financial investment and gambling are inherently diverse constructs, but also that the speculative component of financial trading shares similarities with gambling, and that stock market speculation and problem gambling in a society therefore may be correlated (10).

One type of stock market trading is the so-called “day trading,” characterized by a frequent and often continuous and rapid investment pattern aimed to gain money from very short-term market activities. Day trading has been reported to be associated with more intense and more problematic gambling habits with respect to traditional gambling types, compared to the general population (11). While day traders may share some characteristics with patients with problem gambling, they also may differ with respect to gambling characteristics, with a higher preference for skill-based games (11). In addition, trading with modern forms of financial instruments, such as cryptocurrencies, has been increasingly highlighted, as a potentially addictive type of gambling behavior. This has been shown to be associated with other stock market activity and with a higher degree of gambling problems (12).

## COVID-19 and Potential for Increased Day Trading Behavior and Debts

Several behavioral changes and changes in mental health can be suspected to occur during the COVID-19 pandemic and its impact on everyday life, including the increase in time spent at home, job insecurity, and concerns about one's future financial situation. This has called for research attention to a range of online-based addictive behaviors, such as online gaming (13), online gambling (5), and online pornography use (14). In addition, financial insecurity, job insecurity or sudden unemployment may have caused substantial financial and psycho-social problems during the pandemic. For example, many settings saw a rapid rise in unemployment rates during the first phase of the pandemic (15). In addition, the COVID-19 crisis has been the cause of most volatile financial markets globally, in particular during the first phases of the pandemic (16).

During the COVID-19 pandemic, rapid, online investment services have been increasingly discussed in the context of addictive gambling. Day trading intuitively shares characteristics with traditional gambling, and typically with the rapid-outcome, online-based gambling types which are either entirely chance-based or involving some degree of skills (17). This change also has been reported, in media reports, to include an increase in treatment seeking for gambling disorder among individuals involved in day trading, for example caused by more time working from home (18). In addition, stock exchange trading in general has been described to increase as a consequence of the COVID-19 pandemic (19). Moreover, during the very first phase of the pandemic, sports events globally were canceled, and it has been shown that this affected the distribution of gambling across gambling types, favoring non-sports-related or horse-related betting practices (20). In line with these reports of a certain migration between traditional gambling types, media have also reported that people with intense gambling conditions may have turned to stock day trading as an equivalent behavior to gambling (21). While this topic is concerning and discussed in the media, there is hitherto limited scientific reporting about the extent and nature of day trading behaviors during the pandemic, and about its association to gambling and disordered gambling. This includes the need for scientific data describing whether day trading practices may have recruited new clients or whether it has increased primarily in clients engaging in these practices before, or whether it has recruited new individuals, for example as a sign of migration from traditional gambling types. Related to this, there is need for research-based data addressing whether the providers of day trading opportunities may have adapted to the COVID-19 situation and to the opportunity to recruit new groups of gamblers. Given the similarity of day trading apps with gambling sites (17), it is less known how marketing strategies may have adapted to the COVID-19 crisis. Thus, the line of research addressing gambling advertising and its impact on gamblers, may need to expand to the marketing of financial instruments, including in times of the pandemic and similar crises.

## Call for Research and Clinical Guidelines

Although increasingly understood as a gambling-like behavior, with the potential to cause a gambling disorder, day trading is sparsely addressed in the mental health literature. As media reports indicate a relatively pronounced day trading behavior during the COVID-19 pandemic, preventive and therapeutic interventions are likely to be needed. Also, given the impact of indebtedness on mental health, there is reason to believe that the present type of stock trading behavior may contribute to mental distress during and beyond the pandemic (7).

Assessment instruments for problem gambling should include stock exchange and similar as a gambling type, allowing for the study of treatment seeking and for the monitoring of longitudinal changes in the gambling types reported by problem gamblers in the general population. For example, Addiction Severity Index-Gambling (ASI-G), even in its earliest versions, includes this kind of non-traditional stock market data in the assessment of gambling problems (22), although such data are rarely reported. Thus, although this represents a minority of treatment-seeking individuals with gambling problems, the reporting of stock market use as a gambling problem may need to be followed over time, and even more so in light of the COVID-19 pandemic and its consequences on everyday working habits and financial transactions. In addition to research needs, clinical guidelines are likely also needed, and even more so in response to the current COVID-19-related changes. In particular, socio-demographic characteristics in people with addictive day trading habits may differ from those of patients with more traditional gambling problems. Likewise, within the group of day traders with addictive patterns, it is of value to study whether there are subgroups, for example traders with or without parallel traditional gambling practices. Such subgroups may differ with respect to personality features or other comorbidities, and given the lack of such data so far, such research is warranted and may have clinical implications.

## Discussion

The present paper highlights a topic rarely assessed in the scientific literature concerning gambling, although it may lead to severe financial problems through the same mechanisms as a more traditional gambling behavior, and thereby to severe health concerns. Mental health settings, including gambling disorder treatment facilities, should actively assess and monitor stock market trading, including day trading, in their patients. In addition, credit counselors, and social service staff involved in indebtedness issues, may need to address stock market trading from the same perspective as a more traditional gambling problem involving, for example, sports betting and casino gambling. Worrying media reporting about an increased day trading behavior during COVID-19 raises the challenge to take rapid action in the active monitoring and prevention of addictive day trading behaviors, over-indebtedness and poor mental health.

## Data Availability Statement

The original contributions presented in the study are included in the article/supplementary material, further inquiries can be directed to the corresponding author.

## Author Contributions

All authors were responsible of the overall research idea, its theoretical background, and the message of the paper. AH wrote the manuscript draft, and all authors contributed with input and revision of the manuscript and approved its final version.

## Conflict of Interest

AH has a researcher position at Lund University, sponsored by the state-owned gambling operator of Sweden (AB Svenska Spel). He also has research funding from the research councils of the same state-owned gambling operator and the Swedish alcohol monopoly (Systembolaget) and the Swedish Sport Federation. None of these organizations were involved in the present research, and did not have any influence on it. FF-A reports consultation fees from Novo Nordisk and editor-in-Chief honorarium from Wiley. The remaining author declares that the research was conducted in the absence of any commercial or financial relationships that could be construed as a potential conflict of interest.

## Publisher's Note

All claims expressed in this article are solely those of the authors and do not necessarily represent those of their affiliated organizations, or those of the publisher, the editors and the reviewers. Any product that may be evaluated in this article, or claim that may be made by its manufacturer, is not guaranteed or endorsed by the publisher.
